# The presence of *Ixodes pavlovskyi* and *I. pavlovskyi*–borne microorganisms in Rishiri Island: an ecological survey

**DOI:** 10.1128/msphere.00213-23

**Published:** 2023-11-06

**Authors:** Aya Zamoto-Niikura, Akiko Saigo, Masahiko Sato, Hirotaka Kobayashi, Mizuki Sasaki, Minoru Nakao, Tadaki Suzuki, Shigeru Morikawa

**Affiliations:** 1 Management Department of Biosafety, Laboratory Animal and Pathogen Bank, National Institute of Infectious Diseases, Tokyo, Japan; 2 Rishiri Town Museum, Hokkaido, Japan; 3 Department of Pathology, National Institute of Infectious Diseases, Tokyo, Japan; 4 Asahikawa Medical College, Hokkaido, Japan; 5 Department of Veterinary Medicine, Okayama University of Science, Okayama, Japan; University of Wisconsin–Madison, Madison, Wisconsin, USA

**Keywords:** *Ixodes pavlovskyi*, tick-borne, wild bird, island ecology, Piroplasmida, Ehrlichia, *Babesia microti*, rodent-borne

## Abstract

Rishiri Island, towering toward the Sea of Japan, has been volcanically dormant for approximately 8,000 years. This small inhabited island is free of middle- to large-sized wildlife and a crucial stopover for wild birds migrating along various routes of the East Asian Flyway. A 5-year survey was conducted to explore the biogeography of ticks and tick-borne microorganisms. By flagging vegetation, *Ixodes pavlovskyi* (Pomerantzev, 1948), distributed in limited spots in the Far East, was predominantly collected (60%–80% collection) throughout the survey period. The *I. pavlovskyi* consisted of two haplogroups, Asahikawa-type and Rishiri-type, with the prevalence and nucleotide diversity of Rishiri-type being over 90% and 0.068, respectively. A survey of wild animals revealed that red-backed voles and wild birds, including Oriental greenfinches and black-faced buntings, are their hosts for blood feeding. Furthermore, the red-backed voles were infected with tick-borne *Candidatus* Ehrlichia khabarensis (5/21, 24%). Till date, microorganisms with identical gene sequences have only been reported from Khabarovsk and Vancouver. *Ca*. E. khabarensis gene has also been detected in host-seeking adult *I. pavlovskyi*. These results indicated that Rishiri Island is a refuge for both *I. pavlovskyi and I. pavlovskyi*–borne microorganisms. Additionally, the *Babesia microti* US lineage, which is vectored by *Ixodes persulcatus* in the Far East, appears to be maintained between *I. pavlovskyi* and wild rodents. Various factors have influenced the unique ecosystem of the island. The historical and ecological biogeography of Rishiri Island helps us understand the origin, evolution, and expansion of ticks and associated microorganisms.

**IMPORTANCE:**

Understanding the ecology of ticks and tick-borne microorganisms is important to assess the risk of emerging tick-borne diseases. Despite the fact that the *Ixodes pavlovskyi* tick bites humans, we lack information including population genetics and the reason for the inadequate distribution in Japan. A 5-year survey revealed that Rishiri Island, the main stopover in the East Asian Flyway of wild birds in the northern Sea of Japan, was a refuge of *I. pavlovskyi*. The *I. pavlovskyi* included two haplogroups, which were supposed to diverge a long time before the island separated from the continent and Hokkaido mainland. The detection of microorganisms from wildlife revealed that wild birds and rodents play a role in diffusion and settlement, respectively, of not only *I. pavlovskyi* but also *I. pavlovskyi*–borne microorganisms including *Candidatus* Ehrlichia khabarensis and *Babesia microti* US lineage. Various island-specific factors control *I. pavlovskyi* dominance and tick-borne pathogen maintenance. The results may enable us to explain how tick-borne infectious microorganisms are transported.

## INTRODUCTION

*Ixodes pavlovskyi* Pomerantzev, 1948 has been actively collected in the Palearctic region, which is geographically discontinued from Western Siberia (Altai Mountains and Novosibirsk) to the Far East (Island Russky in Primorskaya Oblast and Japan) (1–3). Additionally, *I. pavlovskyi* was recorded from the repository in China: females from Blue Hill Pigeon *Columba rupestris* in Manasi, Xinjiang autonomous regions (1973), and adults, nymphs, and larvae in bird nests in Dunhua County, Jilin province (1959) (4). *Ixodes persulcatus* is always sympatrically distributed in the habitat area. These species are not only morphologically similar but also genetically closely related and partly transmit the same pathogens, including tick-borne encephalitis virus, *Borrelia* spp., and *Rickettsia* spp. (5–7).

**Fig 1:**
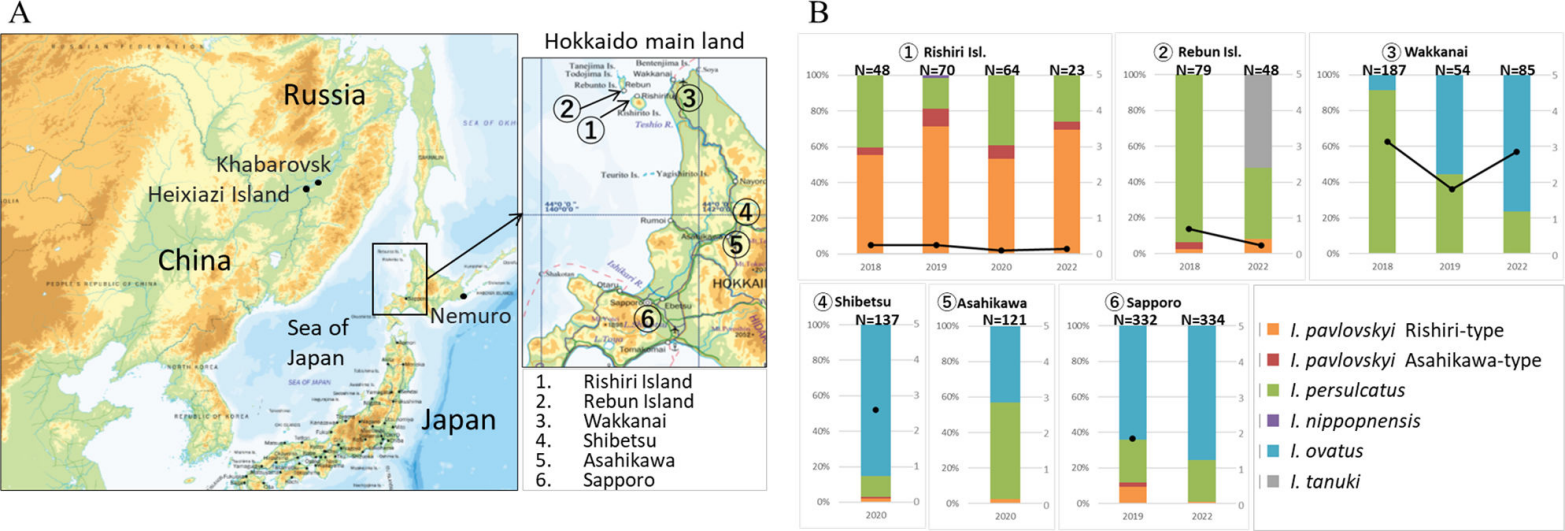
Survey sites and tick collection by flagging vegetation on Rishiri Island and other areas in May or June. (**A**) Map of Hokkaido, Japan (partial). The number on the map shows the location where ticks were collected. (**B**) Bar and line charts show the proportion of tick species (left *y*-axis) and tick collection efficiency (tick/min) (right *y*-axis), respectively. The number below each bar indicates the year collected. Source: topographic map by Geospatial Information Authority of Japan (https://maps.gsi.go.jp/#5/36.120128/140.097656/&base=english&ls=english&disp=1&vs=c1g1j0h0k0l0u0t0z0r0s0m0f1&d=m).

The distribution of *I. pavlovskyi* in Japan was first confirmed in 1992 in Asahikawa and the area surrounding Hokkaido mainland (8) (Fig. 1). Approximately 0.4%–4％ of *Ixodes* ticks collected from the habitat area are *I. pavlovskyi* (Nakao personal communication). Tick species are difficult to distinguish from the dominant sympatric *I. persulcatus*; thus, their life cycle, genetic structure, and importance as vectors for tick-borne diseases have been scarcely studied (9).

Rishiri Island (N45°10′, E141°14′) is the largest island (183 km^2^) in Hokkaido, located at the northernmost tip of Japan, and about 40 km southwest of Wakkanai city (Fig. 1). Rishiri Island is a volcanic island that has been volcanically dormant for approximately 8,000 years (10). Mt. Rishiri is a 1,721 m high stratovolcano located at the center of Rishiri Island. Thus, the island is a landmark for many migratory birds that fly between Eurasia and Japan. To date, more than 326 migratory bird species have been identified on this island (11). Additionally, wild mammals on Rishiri Island are unique because weasels are the biggest wildlife, and middle- to large-sized animals, including bears, deer, foxes, and raccoon dogs, are absent.

In 2018, we surveyed ticks by flagging vegetation on Rishiri Island and detected morphologically ambiguous *Ixodid* male and female ticks similar to *I. persulcatus* males and *Ixodes ovatus* females, respectively. Subsequently, the ticks were confirmed as *I. pavlovskyi* based on key factors described by Nakao et al. (8) and mitochondrial cytochrome c oxidase subunit I (*COI*) gene sequences (12). Interestingly, we found that the *I. pavlovskyi* population was genetically diverse (maximum 1%), and the difference was significant for classifying them into two sub-lineages, namely, Asahikawa-type and Rishiri-type (13) (Supplement S1).

To unveil the ecology of *I. pavlovskyi*, we performed a 5-year survey, including tick collection from various wild animals, molecular analysis, cross-breeding, and genetic isolation of vector-borne pathogens. The results obtained in this study suggest that various island-specific factors control *I. pavlovskyi* dominance, vector-borne pathogen maintenance, and disproportionate distribution of both *I. pavlovskyi* haplogroups over Rishiri Island and the Hokkaido mainland.

## MATERIALS AND METHODS

### Field collection

From 2018 to 2022, unfed, host-seeking ticks were collected in April and May from Rishiri Island and the neighboring Rebun Island, 10 km north of Rishiri Island, by flagging vegetation on the sides of forest paths easily accessible by car and on foot (Fig. 1A). Ticks were also collected from the Hokkaido mainland. Wakkanai is located approximately 40 km northeast of Rishiri Island. The ticks in Shibetsu, Asahikawa, and Sapporo have also been investigated since *I. pavlovskyi* was first recorded (8, 9). The ticks were initially classified morphologically, based on descriptions by Nakao et al. and Takada (8, 14). Female and male ticks were kept alive separately in tightly sealed plastic tubes over a moistened filling of solidified plaster with activated charcoal at 4℃ until mating. Other ticks were frozen and stored at −30°C until DNA extraction. Tick collection efficiency was used to determine how often tick-carrying wild animals appeared at the survey site (tick/min/person) (15). Several people collected ticks from Asahikawa and Sapporo in 2022, and the collection efficiency was not calculated. Ticks collected in 2018 on Rishiri Island were originally used in Zamoto-Niikura et al. (13).

Small wild mammals were collected on Rishiri Island in 2020 and 2022 using small snap traps (Panchu PMP, HOGA, Kyoto, Japan) and Sharman live traps, respectively. The animal species were identified morphologically (shrews and rodents) and molecularly (rodents) based on the cytochrome b gene (*cyt-b*) sequence (16). Ticks feeding on wildlife were manually removed. The ticks almost engorged were reared in the laboratory at 20°C to hatch or lay eggs. DNA was extracted from the resulting ticks and used for the detection of microorganisms by PCR as described below (transstadial transmission). The permissions required for the survey were obtained under the Wildlife Protection, Control, and Hunting Management Act. The ticks in road-killed animals including birds, weasels, and cats were also investigated.

### DNA extraction from ticks and rodents

DNA was extracted from ticks by a standard protocol using the phenol/ethanol method (15). The spleens of the wild rodent were shred to 100 mg pieces and homogenized in a tube containing a ceramic ball (Ceramic Sphere, MP Biomedicals; catalog no. 6540412), beads (Garnet Matrix A Bulk, MP Biomedicals; catalog no. 6540–427), and TNE buffer (50 mM Tris–HCl [pH 7.4], 100 mM NaCl, and 0.1 mM EDTA) by shaking at 4,000 rpm for 30 s (Micro Smash MS-100, TOMY). Proteinase K (0.1 mg/mL) and SDS (0.1%) were added and incubated at 55°C overnight. DNA was extracted using the standard phenol/ethanol method, dissolved in 200 µL TE (10 mM Tris–HCl, pH 7.4, 1 mM EDTA), and stored at −30°C.

### Establishment of a PCR–restriction fragment length polymorphism (RFLP) system for live tick identification

**Fig 2:**
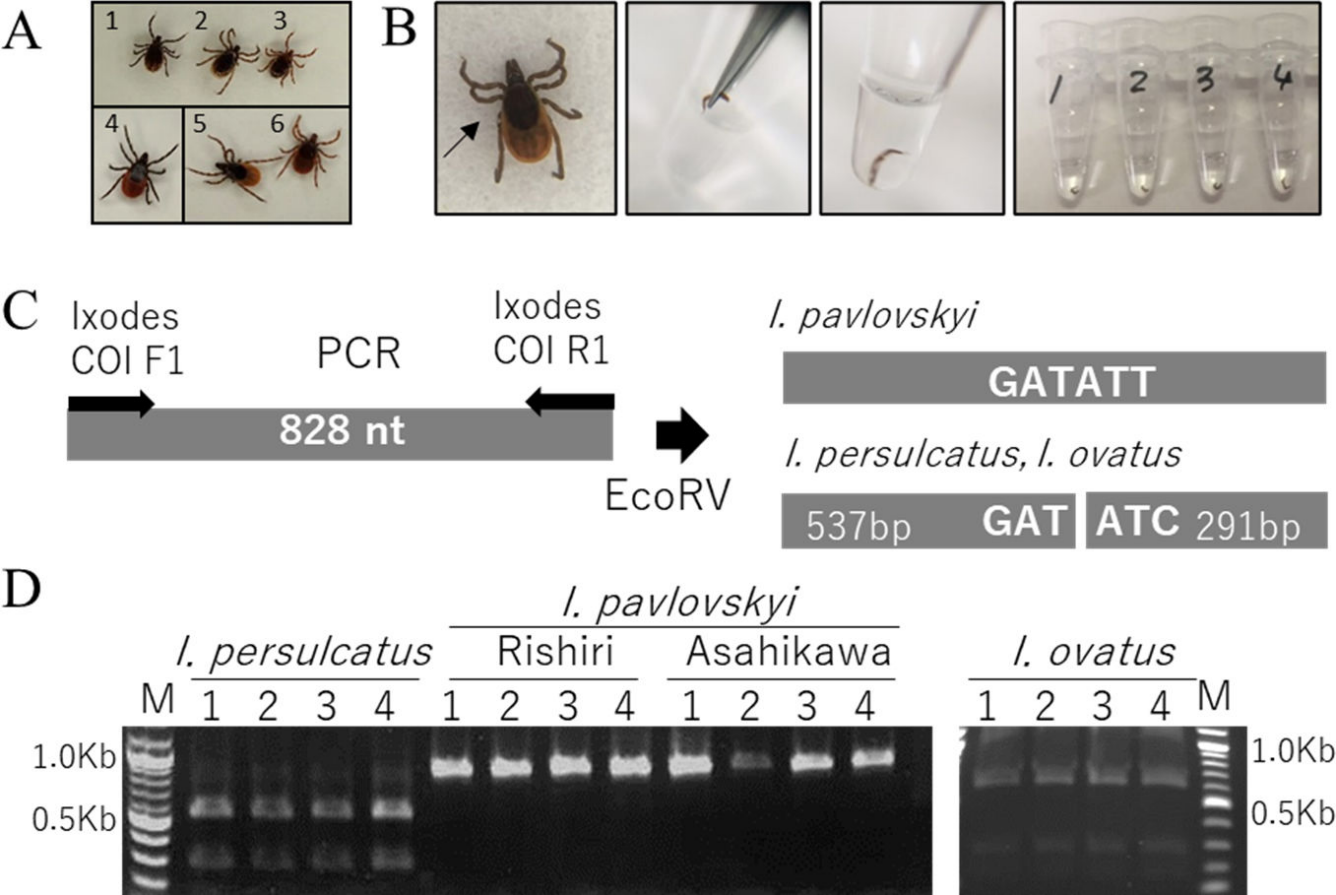
Identification *of Ixodes pavlovskyi* by PCR–RFLP. (**A**) Images *of I. persulcatus, I. pavlovskyi,* and *I. ovatus* (left to right). Upper and lower panels show males and females, respectively. (**B**) Direct PCR using a tick leg cut and directly submerged into PCR mixture. (**C**) Schematic diagram of the amplification primers and EcoRV recognition site. (**D**) Electrophoresis after PCR–RFLP of *I. pavlovskyi, I. persulcatus,* and *I. ovatus*. Two haplogroups, Rishiri-type and Asahikawa-type, of *I. pavlovskyi* are used. M, DNA size markers.

To distinguish live *I. pavlovskyi* from *I. persulcatus* and *I. ovatus*, we developed a PCR–RFLP method based on hints from the hemolymph test (17, 18). “Leg II” or “leg III” of one side was amputated using ophthalmic scissors and directly added to the PCR mixture as the template (Fig. 2). All ticks with an amputated leg were reared in moistened tubes at 4°C. All ticks were alive (data not shown). PCR was performed using the Phusion Blood Direct PCR Kit (Thermo Fisher Scientific; catalog no. F547S). The thermal cycling program was set to the annealing temperature at 55°C and extension time of 1 min. The primer set, *Ixodes* COI F1/Ixodes COI R1 (Supplement S2), was designed to amplify the mitochondrial *COI* sequence of various *Ixodes* spp. collected from the Hokkaido mainland and Rishiri Island. Sequence diversity among *Ixodes* spp. was used to design an RFLP assay (Table 1).

**TABLE 1:** Unique restriction enzymes and expected fragment sizes of *COI* amplicons (828 nt)^*a*^^,^^*b*^

Species of tick^*c*^	Restriction enzymes			
	*Eco*RV	*Sca*I	*Xho*I	*Hpa*I
*I. pavlovskyi*	(−)	(−)	545, 283	(−)
*I. persulcatus*	537, 291	668, 160	545, 283	(−)
*I. ovatus*	537, 291	(−)	(−)	704, 124
*I. turdus*	(−)	(−)	(−)	(−)
*I. nipponensis*	(−)	(−)	(−)	(−)
*I. tanuki*	(−)	(−)	545, 283	(−)
*I. signatus*	(−)	652, 176	545, 283	704, 124
*I. angustus*	(−)	668, 160	(−)	(−)

^*a*^ PCR products (828 nt) are amplified by using the primers Ixo COI F1 and Ixo COI R1 (Supplement S2).

^*b*^ Absence of recognition site is indicated as (−).

^*c*^ Accession numbers of reference *COI* sequences are as follows: *I. pavlovskyi*, AB231669; *I. persulcatus*, AB073725; *I. ovatus,* AB231670; *I. turdus*, AB231668; *I. nipponensis*, AB231671; *I. turdus,* MT371811; *I. tanuki,* MT371810; *I. angustus,* LC718586.

### Molecular identification of wild rodents

The mammalian *Cyt-b* gene (16) (Supplement S2) was amplified from spleen DNA by using Ex Taq (Takara; catalog no. RR001A) according to the manufacturer’s instructions. The PCR amplicons were directly sequenced using the primers used for amplification (Eurofins Genetics, Tokyo, Japan).

### Population genetics

The *COI* sequence-based haplotypes (partial 701 bp) were determined using DnaSP version 6.12.03 (http://www.ub.edu/dnasp/), and the network was constructed using the median-joining method in the NETWORK 10.2.0.0 program (https://www.fluxus-engineering.com/). In total, 104 sequences from *I. pavlovskyi*, 84 sequences from *I. persulcatus,* and a *COI* sequence from *I. pavlovskyi* from Tomsk (accession number JX288763) were used. The fixation index (FST) and other values related to genetic diversity were calculated using DnaSP version 6.12.03.

### Cross-breeding of ticks

Male and female ticks were kept in different containers immediately after collection. After the species and haplogroups were identified, designated males and females were kept in a clear-colored tube at room temperature (20–30°C), and mating was visually confirmed. After the male detached from the female, the female was allowed to feed on a gerbil. Engorged females were reared at 20°C until egg laying. The eggs were aliquoted to small glass tubes and incubated at 20°C. The larvae hatched from the eggs approximately 1 month later. If the larvae did not hatch from the egg, they were kept in a container for 6 months for extended observation.

### Whole mitochondrial genome sequencing of *I. pavlovskyi*

The full-length mitochondrial genome sequence of both haplogroups was determined using a combination of long PCR and Illumina short-read sequencing. Genomes were extracted from eggs laid by *I. pavlovskyi* females and used as templates. Primers were designed to anneal the conserved region of the mitochondrial genome of *Ixodes* (Supplement S2). Two PCR products were obtained using the primer sets IxoMt_18F/IxoMt_9356R or IxoMt_8326F/IxoMt_3458R and Gflex polymerase (Takara). Both fragments were used for library construction (TruSeq Nano DNA Kit, Illumina). Sequencing was performed by using Novaseq6000 (Macrogen, Japan) (150 bp paired end, 4 GB). The sequences were assembled using Bowtie2 version 2.3.4.1.

### Bacterial detection

Anaplasmataceae were detected in the DNA extracted from rodents and ticks using nested PCR targeting 16S rRNA and *groEL* (19) (Supplement S2). Primers for *Bartonella taylorii gltA* amplification were designed in this study (Supplement S2). Ex Taq polymerase (Takara Bio, Tokyo, Japan) was used according to the manufacturer’s instructions. All PCR amplicons of the expected size were directly sequenced (Eurofins Genetics, Tokyo, Japan).

### Detection of *Babesia microti* from rodents

Blood samples were processed as described previously (20). Blood smears were used for microscopic examination of intraerythrocytic protozoa. DNA extracted from rodent spleens was examined using nested PCR targeting Piroplasmida *18S rRNA* and *Babesia microti* Chaperonin Containing TCP1 Subunit 7 (*CCT7*) (21, 22) (Supplement S2). Nested PCRs and direct sequencing were performed as described previously (15).

### Sample preparation for scanning electron microscopy (SEM)

The ticks were fixed with 70% ethanol. After dehydration using an ascending series of ethanol solutions, the specimens were air-dried. After mounting on aluminum stubs with carbon paste, the dried specimens were coated with osmium using an osmium plasma coater (Neoc-ST; Meiwafosis, Tokyo, Japan) and observed under a scanning electron microscope (SU6600; Hitachi, Tokyo, Japan).

### Experimental animals

Pathogen-free Mongolian gerbils (MON/Jms/Gbs) were purchased from SLC (Shizuoka, Japan). The animal experiments were performed in accordance with the Laboratory Animal Control Guidelines of the National Institute of Infectious Diseases (215038).

## RESULTS

### Identification of *I. pavlovskyi* by PCR–RFLP

The distribution, habitat, and active season of *I. pavlovskyi* overlapped with those of *I. persulcatus* and *I. ovatus*, both of which are predominantly found in Hokkaido (mainland). Furthermore, they are morphologically similar (Fig. 2A). To avoid misclassification, we developed a PCR–RFLP method based on the *COI* gene of *Ixodes*. The various restriction enzyme recognition sites and the expected fragment sizes after treatment are summarized in Table 1. Figure 2B shows the PCR procedure using a tick leg as the template. PCR amplification and EcoRV digestion (Fig. 2C) distinguished *I. pavlovskyi* from *I. persulcatus* and *I. ovatus* (Fig. 2D), regardless of the sequence type (13) (Supplement S1, phylogenetic tree of *I. pavlovskyi* and *I. persulcatus*). Both male and female ticks survived after removing the legs individually, and some were subsequently mated. We also screened the specimens collected previously from the eastern part of Hokkaido (15, 23) and did not detect *I. pavlovskyi* (data not shown). Hybrid of *I. pavlovskyi/I. persulcatus* was recently described from Siberia (24) and the Far East (3). The molecular identification of *I. pavlovskyi* based on a chromosomal gene, *toll* (25) (Supplement S8), did not detect the hybrids, although species identification should be carefully performed on the ticks collected in sympatric areas (24, 25).

### SEM

**Fig 3:**
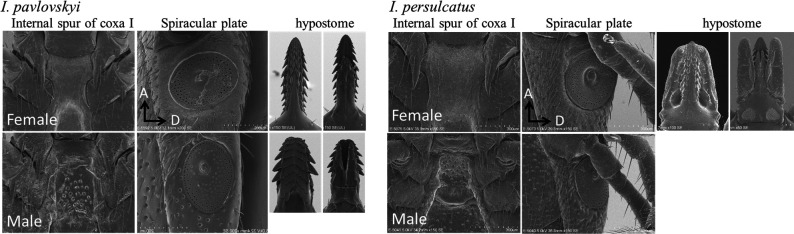
Internal spur of coxa I, spiracular plate and hypostome *of Ixodes pavlovskyi* and *Ixodes persulcatus*. F, female; M, male; A, anterior; D, dorsal.

The internal spur of coxa I and the spiracular plate of adult *I. pavlovskyi* (Rishiri-type) are shown in Fig. 3. The internal spur of coxa I in both males and females was shorter than that of *I. persulcatus*. The spiracular plates of *I. pavlovskyi* are oval. These keys correspond to those proposed by Nakano et al. The apices of the hypostomes of *I. pavlovskyi* and *I. persulcatus* females were similar, although they were described to be acute and round at the apex, respectively (Supplement S5). Other organs are shown in Supplement S5.

### *I. pavlovskyi* was predominantly collected by flagging vegetation on Rishiri Island

Collection sites of adult host-seeking ticks are shown in Fig. 1A. *I. pavlovskyi* always accounted for 60%–80% of ticks collected from Rishiri Island (Fig. 1B). The ratio of the Rishiri-type to Asahikawa-type ticks was approximately 9:1. Collection efficiency was 0.08 tick/min (2020) to 0.24 tick/min (2018). *I. pavlovskyi* was collected from Rebun Island (no. of *I. pavlovskyi*/no. of collection, 5/79 and 4/48 in 2018 and 2022, respectively), Shibetsu (4/137), Asahikawa (3/121), and Sapporo (39/332 and 3/334 in 2019 and 2022, respectively). The Asahikawa-type was collected sympatrically with the Rishiri-type from all *I. pavlovskyi* collection sites. *I. pavlovskyi* was not collected from Wakkanai. The low prevalence of *I. pavlovskyi* in Asahikawa and Sapporo and their absence in the northern areas, such as Wakkanai, corresponded to previous observations (9, 26).

### Haplotype network of *I. pavlovskyi* revealed a structured population

**Fig 4:**
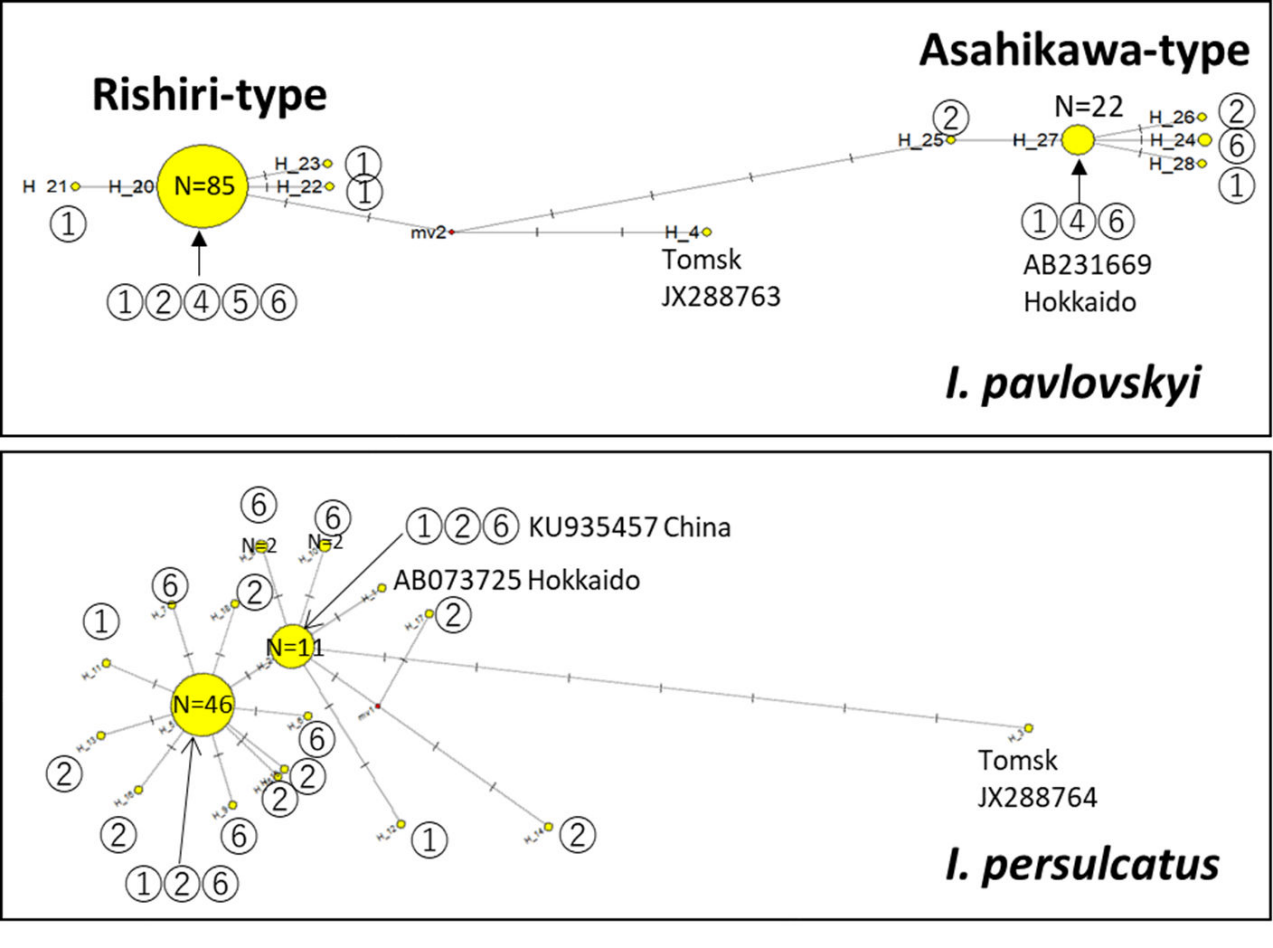
Haplotype network based on the partial *COI* sequence (701 bp). Haplotypes were determined using DnaSP, and the network was constructed by the median-joining method in the NETWORK 10.2.0.0 program. The number on the network shows the location where ticks in each haplotype were collected: (1) Rishiri Island, (2) Rebun Island, (3) Wakkanai, (4) Shibetsu, (5) Asahikawa, and (6) Sapporo.

A haplotype network based on *COI* sequences of *I. pavlovskyi* collected from five areas (Fig. 1B) (*n* = 114) and a reference from Tomsk is shown in Fig. 4. Two distinct haplogroups, Rishiri and Asahikawa types, were clearly identified, regardless of the geographic origin of the ticks (Fig. 1A). These haplogroups correspond to the two lineages in the phylogenetic tree (13) (Supplement S1). Among the 114 *I. pavlovskyi* examined, 88 (77%) were of Rishiri-type and 97% (85/88) were identical (H_20). Asahikawa-type (22.8%, 26/114) was a minor haplogroup. Almost all (22/26, 84.7%) were of haplotype H_27. Both types were distinct from the reference sequence from Tomsk (JX288763). The haplotype network of *I. persulcatus* (*n* = 84) did not exhibit a clear structure.

### Rishiri-type and Asahikawa-type ticks are genetically distinct

To investigate the degree of genetic differentiation between the Rishiri and Asahikawa types, the genetic differentiation index (FST) was determined based on all *COI* sequences used in the haplotype network (Table 2). In all comparisons, the FST values were higher than 0.95. In particular, the FST value between the Rishiri and Asahikawa types was 0.95316, suggesting substantial genetic differentiation.

**TABLE 2:** Genetic diversity between *I. pavlovskyi* Rishiri-type and Asahikawa-type and *I. persulcatus*

Population 1	Population 2	FST
*I. pavlovskyi* Rishiri-type	*I. pavlovskyi* Asahikawa-type	0.95316
*I. persulcatus*	*I. pavlovskyi* all	0.97536
*I. persulcatus*	*I. pavlovskyi* Rishiri-type	0.99145
*I. persulcatus*	*I. pavlovskyi* Asahikawa-type	0.98709

The genetic diversity of all tick populations is shown in Table 3. The haplotype diversity (Hd) of the Asahikawa-type and *I. persulcatus* was higher than 0.5, whereas that of the Rishiri-type was 0.067. The nucleotide diversity (Pi) of the Rishiri-type (approximately 0.0001) was one-tenth of that of the Asahikawa-type and *I. persulcatus*, suggesting that the Rishiri-type has been structured recently and that genetic variation among the Rishiri-type populations has not yet accumulated.

**TABLE 3:** Genetic diversity and selection pressure

	No. of sequences	No. of nucleotide mutations		Diversity indexes					Neutrality	
		Synonymous	Non-synonymous	No. of segregating sites (S)	No. of haplotypes (h)	Haplotype diversity (Hd)	Average no. of differences (K)	Nucleotide diversity (Pi)	Tajima’s *D*	Fu’s Fs
*I. pavlovskyi*										
All	104	12	1	13	9	0.323	1.961	0.0028	−0.57182	−0.172
Rishiri-type	88	2	1	3	4	0.067	0.068	0.0001	−1.62640	−5.444
Asahikawa-type	16	4	0	4	5	0.533	0.608	0.0009	−1.54972	−2.751
*I. persulcatus*	84	16	2	18	16	0.632	0.932	0.0013	−2.16120^*a*^	−13.854

^*a*^ Significant (*P* < 0.05).

### Complete mitochondrial DNA sequence of Rishiri-type and Asahikawa-type

The complete mitochondrial DNA of the Rishiri-type (LC578482) and Asahikawa-type (LC633335) were 14,575 nucleotides (nt) long and circular, which corresponded to that of *I. pavlovskyi* Tms12-02 strain from Tomsk (KJ00060). Moreover, the gene organization of the Rishiri- and Asahikawa-type mitochondrial genomes was the same as that of Tms12-02. Comparing the full-length mitochondrial DNA showed 142, 189, and 255 nt differences between Rishiri- and Asahikawa-type, Rishiri-type and TMS 12-02, and Asahikawa-type and TMS 12-02 populations, respectively (Supplement S6). *COI* of OL741747, *I. pavlovskyi* mitochondrion genome from Hokkaido, Japan (14,559nt), was identical to that of Rishiri-type H_20 (Fig. 4). Percent identities between OL741747 and either of LC578482, LC633335, and KJ00060 were 99.77%, 98.91%, and 98.26%, respectively.

### Homologous but not heterologous pairs produced offspring

**Fig 5:**
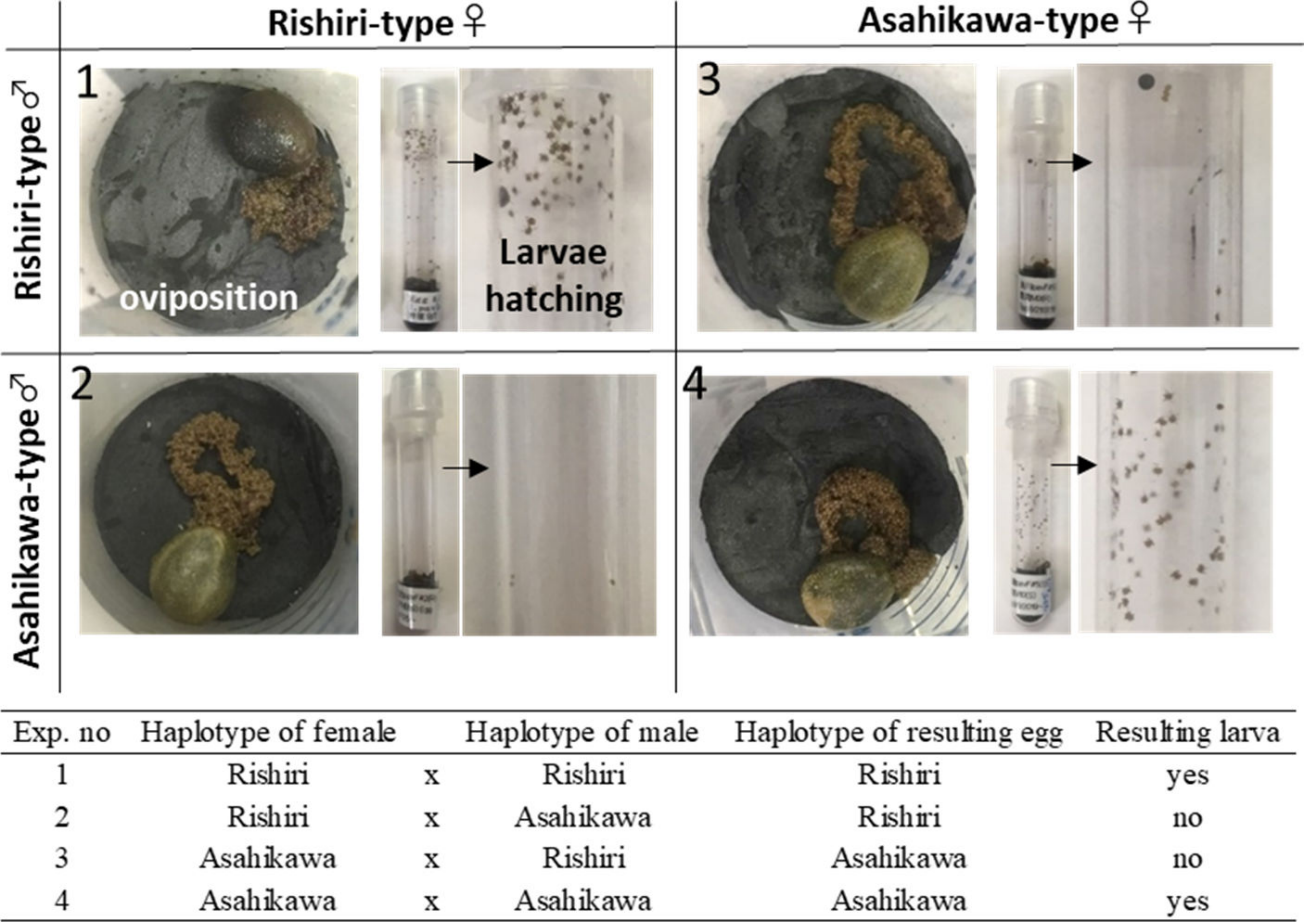
Cross-breeding *Ixodes pavlovskyi* Rishiri-type and Asahikawa-type. The images on the upper panel show females laying eggs and larvae hatched from the eggs. Female ticks of both types mated with the same or different types of male ticks are shown. The lower panel shows the summary of the cross-breeding experiment.

Genetic differentiation between the two types was high (Table 2), and both types were collected sympatrically during the field examination (Fig. 1). These data indicate that reproductive isolation may occur. To test the biological difference, we tried to conduct a cross-breeding experiment by mating homologous and heterologous pairs (Fig. 5). The feeding period (approximately 7 d) and initiation of egg laying (approximately 1 month) did not differ among females tested (data not shown). However, larvae hatched from eggs within 3 months only when the homologous pairs were mated. The experiment (four pairs) was repeated once and resulted in similar observations. The number of Asahikawa-type was not sufficiently collected, and paired ticks were not always mated. More tests are required to confirm the sterility.

### *I. pavlovskyi* fed on wild rodents and migrating birds in spring

The ticks collected from wild animals are shown in Table 4. *I. pavlovskyi* of all stages fed on wild birds, including the Oriental Greenfinch (*Chloris sinica*) and black-faced bunting (*Emberiza spodocephala*) (Supplement S7). Immature *I. pavlovskyi* was also found on red-backed voles captured in May. *I. pavlovskyi* was completely absent from wild rodents captured in June and October. During these months, *I. angustus* and *I. tanuki* were major ticks.

**TABLE 4:** Collection of ticks from animals in Rishiri Island, 2018–2022

Date	Host species		*I. pavlovskyi*		*I. persulcatus*	*I. angustus*	*I. tanuki*	*I. signatus*	*I. turdus*
			Rishiri-type	Asahikawa-type					
20 May 2020	*Chloris sinica* (*n* = 1)^*a*^	Oriental greenfinch	Female 1	-	-	-	-	-	-
22 May 2022	*Emberiza spodocephala* (*n* = 1)	Black-faced bunting	Nymph 1	Larva 2	Nymph 2	-	-	-	-
31 May 2022	*Myodes* spp. (*n* = 8)^*b*^	Red-baked vole	Larva 19	Nymph 1	-	Larva 11, nymph 8, female 8	Larva 1	-	-
24 June 2018	*Larus schistisagus* (*n* = 1)	Slaty-backed gull	-	-	-	-	-	Nymph 2, female 2	-
28 June 2018	*Myodes rufocanus bedfordiae* (*n* = 1)	Red-baked vole	-	-	-	Larvae 10, nymph 5, female 2	-	-	-
23 August 2019	*Mustela itatsi* (*n* = 1)	Japanese weasel	-	-	-	-	Nymph 3, female 3	-	-
23 August 2018	*Felis catus* (*n* = 1)	Domestic cat	-	-	-	-	Female 1	-	-
August–October 2021	*Mustela itatsi* (*n* = 3)	Japanese weasel	-	-	-	-	Nymph 28, female 26	-	-
19 October 2020	*Craseomys* spp. (*n* = 4)^*b*^	Red-backed vole	-	-	-	-	Larvae 1, nymph 8	-	-
	*Apodemus speciosus* (*n* = 6)	Large Japanese field mouse	-	-	-	Nymph 1	Larvae 9, nymph 20	-	-
	*Sorex unguiculatus* (*n* = 2)	Long-clawed shrew	-	-	-	-	Nymph 6	-	-

^*a*^ Sato et al. 2021 Rishiri studies, (40): 25–28.

^*b*^ *Myodes rex* and *M. rufocanus bedfordiae*.

### *Candidatus* Ehrlichia khabarensis, *Bartonella taylorii*, and *Babesia microti* were identified in wild rodents

**Fig 6:**
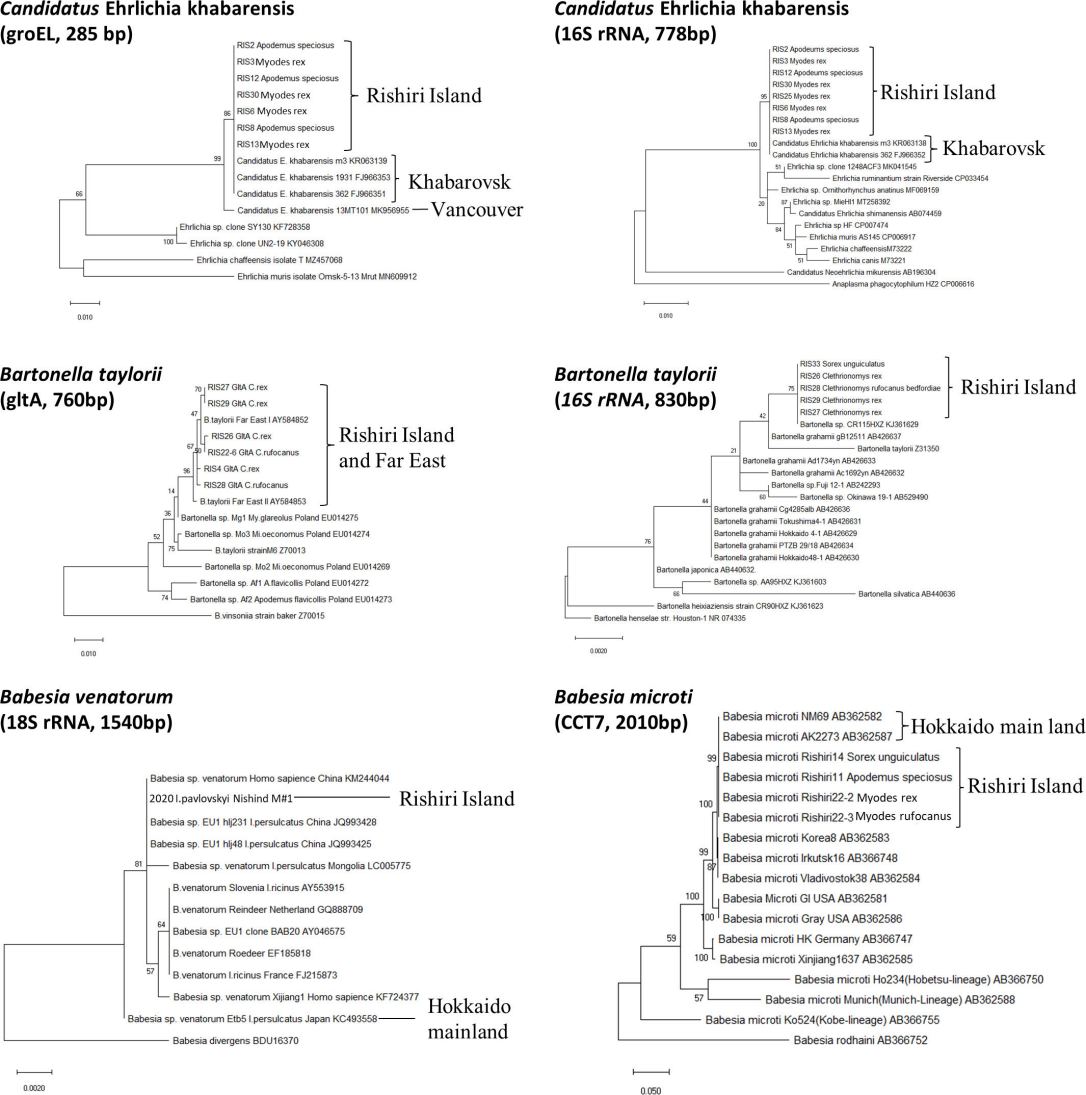
Evolutionary analysis of various microorganisms detected from ticks and rodents. The evolutionary history was inferred using the maximum likelihood method and Tamura–Nei model. The percentage of trees in which the associated taxa clustered together is shown next to the branches. Evolutionary analyses were conducted in MEGA X. Representative samples determined in this study were included in the phylogenetic analyses.

The microorganisms in the spleen of small wild mammals captured in October 2020 and May 2022 (Table 4) were examined by PCR and direct sequencing (Table 5). *M. rex* (*n* = 3), *M. rufocanus bedfordiae* (*n* = 2), and *A. speciosus* (*n* = 3) were positive for Anaplasmataceae *groEL*. All sequences (329 nt) were identical to each other and to that of *Ca*. E. khabarensis strain m3 (KR063139) from Khabarovsk, Russia (27) (Fig. 6 and Table S4). Anaplasmataceae 16S rRNA PCR (830 nt) showed 15 were positive. Of these*,* eight from *M. rex*, *M. rufocanus bedfordiae*, and *A. speciosus* were identical to each other and to that from *Ca*. E. khabarensis strain m3 (KR063138) (27) (Supplement S4). These eight samples corresponded to the *groEL*-positive samples described above. The other seven sequences from *M. rex* (*n* = 2) and *M. rufocanus bedfordiae* (*n* = 5) were identical to those from *Bartonella* sp. CR115HXZ (KJ361629) (Fig. 6) (Supplement S4). CR115HXZ was isolated from rodents on Heixiazi Island (Fig. 1) (28, 29). It is noted that *Bartonella* does not belong to *Anaplasmataceae*; the 16S rRNA gene was amplified.

**TABLE 5:** Detection of vector-borne microorganisms in wild small mammals

Species	No. examined	No. animals infected with					
		*Bab. microti* (US lineage)^*a*^		*Can* E. khabarensis^*b*^		*Bar. taylorii* ^*c*^	
October 2020							
*Myodes rex*	6	0		3	(50.0%)	1	(16.7%)
*M. rufocanus bedfordiae*	7	0		2	(28.6%)	4	(57.1%)
*Apodemus speciosus*	18	2^*d*^	(11.1%)	3^*d*^	(16.7%)	0	
*Sorex unguiculatus*	2	1	(50.0%)	0		0	
May 2022							
*M. rex*	3	3^*e*^	(100%)	0		1^*e*^	(33.3%)
*M. rufocanus bedfordiae*	5	3	(60.0%)	0		1	(20.0%)

^*a*^ Confirmed by *CCT7* and 18S rRNA sequences.

^*b*^ Confirmed by *groEL* and 16S rRNA sequences.

^*c*^ Confirmed by *gltA* and 16S rRNA sequences.

^*d*^ A mixed infection of *Bab. microti* (US) and *Ca.* E. khabarensis was included.

^*e*^ A mixed infection of *Bab. microti* (US) and *Bar. taylorii* was included.

PCR targeting the citrate synthase (*gltA*) gene of *Bartnella* spp. was performed to confirm the presence of *Bartonella* spp. in seven *Bar. taylorii* 16S rRNA-positive rodents. *gltA* amplicons were generated from all seven samples. The sequences (760 nt) were closely related to each other (99.4%–99.8% identical) and those from *Bar. taylorii* strain Far East I (AY584852) (99.7%–99.8% identical) and strain Far East II (AY584853) (99.3%– 99.7% identical) (Fig. 6). These strains were isolated from wild rodents in Khabalovsk (30). It is noted that the partial sequence (272 nt) of the *Bar. taylorii* gltA from *M. rufocanus* (RIS22-6) (Fig. 6 and Table S4) was identical to *Bartonella* sp. Hokkaido 16-3 gltA gene (AB290294) from *M. rufocanus bedfordiae* in Hokkaido (31).

PCR targeting Piroplasmida *18S rRNA* showed that nine samples were positive. All sequences were identical to each other and to those of *Bab. microti* US lineage, including isolate IpSG13-1-2 (LC127369) from *I. persulcatus* on the Hokkaido mainland. PCR targeting *Bab. microti CCT7* gene showed nine samples were positive, all of which were positive for *Piroplasmida* 18S rRNA detected above. The *CCT7* sequences (2,169 nt) were identical to each other and to those of strains IpSG13-1-2 (LC127377) and NM69 (AB362582) from *M. rufocanus* (AB362582.1) (Fig. 6). Both strains were isolated near Nemuro, Eastern Hokkaido, Japan (Fig. 1) (15). Since rodents captured in May 2022 were alive, blood films were made, and intraerythrocytic protozoa were observed in all PCR-positive specimens (0.8%–3.4% parasitemia).

### Transstadial transmission of *Bab. microti* US lineage in *I. pavlovskyi*

From the rodents captured in May 2022**,** 15 engorged ticks, including *I. pavlovskyi* larvae and nymph and *I. angustus* larva and females, were collected from three rodents (Table 6). All rodents tested positive for *Bab. microti,* and one *M. rex* (RIS22-2) was positive for *Bar. taylorii* (mixed infection), as determined by PCR. Larvae successfully hatched from the eggs of all females (*n* = 5). PCR and direct sequencing on resulting ticks revealed that three nymphs and one male contained *Bab. microti* US lineage (Table 6), indicating transstadial transmission. The *Ca*. E. khabarensis or *Bartonella* spp. was not detected.

**TABLE 6:** Detection of *Bab. microti* in resulting ticks

ID	Host species	Microorganism infected	No. of *Bab. microti*–positive/no. of resulting tick			
			*I. pavlovskyi*		*I. angustus*	
			Nymph	Male	Nymph	Egg
RIS 22-2	*M. rex*	*Bab. microti and Bar. taylorii*	2/3	0/0	0/1	0/1
RIS 22-3	*M. rufocanus bedfordiae*	*Bab. microti*	0/1	1/1	0/0	0/1
RIS 22-5	*M. rex*	*Bab. microti*	1/4	0/0	0/0	0/3

### Host-seeking *I. pavlovskyi* Rishiri-type carried *Ca.* E. khabarensis and *Babesia venatorum* genes

The presence of tick-borne microorganisms in host-seeking ticks was investigated using PCR (Table 7). Three and one *I. pavlovskyi* Rishiri-type ticks were positive for Anaplasmataceae *groEL* and Piroplasmida *18S rRNA*, respectively. Sequencing following a BLAST search revealed that three *groEL* sequences (329 nt) were identical to each other and those from *Ca*. E. khabarensis strain m3 (KR063139) and rodents on Rishiri Island in this study (Table 5) (27).

**TABLE 7:** Detection of tick-borne microorganism in host-seeking ticks on Rishiri Island

Species^*a*^	Haplotype	Examined	Microorganisms		
			*Ca.* E. khabarensis	*Bab. venatorum^b^*	*Anaplasma phagocytophilum^c^*
*I. pavlovskyi*	Rishiri-type	94	3	1	0
	Asahikawa-type	11	0	0	0
*I. persulcatus*		38	0	0	1
*I. nipponensis*		1	0	0	0

^*a*^ Only adult ticks were examined.

^*b*^ Determined by 18S rRNA sequence.

^*c*^ Determined by 16S rRNA and *groEL* sequences.

The Piroplasmida *18S rRNA* (1,579 bp) sequence was identical to that of *Babesia* sp. *venatorum* reported from *Homo sapiens* (KM244044) and *I. persulcatus* (JQ993428) in China (32) (Fig. 6). A comparison with *Bab. venatorum* Etb5 (KC493558) from *I. persulcatus* in Hokkaido (23) showed substantial differences (Fig. 6).

Additional investigation of *I. persulcatus* DNA revealed the presence of *Anaplasma phagocytophilum groEL*, which was identical to those reported from *I. persulcatus* in Khabarovsk and Hokkaido (HM366578 and JQ622144, respectively) and *Homo sapiens* in South Korea (CP035303).

## DISCUSSION

This study has four major findings. First, *I. pavlovskyi*, a rare species in Japan, is predominantly distributed on Rishiri Island. Second, the tick population included two haplogroups: the Rishiri-type and Asahikawa-type, which are genetically distinct from a representative gene record from Tomsk. Third, *I. pavlovskyi* feeds on wild birds and rodents, with the rodents carrying *Ca*. E. khabarensis. Since this microorganism has been reported only in Khabarovsk, wild birds and rodents were suggested to play a role in *Ca*. khabarensis diffusion and settlement, respectively. Fourth, we identified new foci of *Babesia microti* US lineage, one of the major tick-borne protozoa in the Northern Hemisphere. This organism is maintained between wild rodents and *I. pavlovskyi*, though *I. persulcatus* is a principal vector in the Far East. The results of this study suggest that Rishiri Island has a suitable climate, hosts, and vegetation for *I. pavlovskyi* colonization and *I. pavlovskyi*-borne microorganism introduction/habitat.

Wildlife on Rishiri Island is unique as it does not include large- to medium-sized predators. *Mustela itatsi* is the largest and most common medium-sized wild species. This animal is an alien species introduced between 1933 and 1935 to eliminate wild rodents (33). Additionally, snakes and hares, which damage birds and woodlands, respectively, are not present. In contrast, wild birds are abundant on the island. Rishiri Island is located within the migration routes of many birds and serves as an important location for stopovers (rest and nourishment) and breeding. A total of 326 species were recorded, with the percentage of birds breeding, wintering, residing, and traveling being 30%, 10%, 10%, and 50%, respectively (34). These specific biological features are advantageous for the life cycle of *I. pavlovskyi*, as *I. pavlovskyi* feeds on birds that collect food on the ground (1, 35). The generalist tick*, I. persulcatus,* has similar seasonal activity and geographical distribution (Fig. 1). However, it feeds on large-to-medium-sized animals (females), and expanding its population on the island is hard. In contrast, *I. pavlovskyi* cannot colonize the Hokkaido mainland, which is overpopulated by the wild sika deer, *Cervus nippon*. It has increased almost twice since 2002, and approximately 690,000 deer inhabit the northern, eastern, and central areas (36). In these areas, *I. persulcatus* and other tick species that favor wild sika deer were densely distributed (Fig. 1) (23). We conclude that Rishiri Island has specific ecological factors required for the dominant colonization of *I. pavlovskyi*, including abundant wild birds, cold climate, negative pressure on sympatric *I. persulcatus*, and isolated woodlands. *I. pavlovskyi* is also dominant in parks in the urban areas of Tomsk. Tick species in city parks have recently been transferred from their original habitats to the Altai Mountains. Similarly, the absence of middle-to-large-sized wildlife and the abundance of wild birds are important factors (37).

Ticks are carried by migrating birds over short and long distances (35, 38). Detection of *Ca*. E. khabarensis gene sequence, which is identical to that reported from Khabarovsk, indicates that the microorganism was carried by birds migrating via the Crossing Sea of Japan route. Rishiri Island, as well as the Hokkaido mainland, is located on the East Asian Flyway, which includes the most diverse flyways worldwide. Although there are individual varieties, most migrating birds that stop over Rishiri Island migrate along two major routes: the Sakhalin–Kuril and the Crossing Sea of Japan routes (39). Passerines including Stejneger’s stonechat (*Saxicola stejnegeri*) (40) and rooks (*Corvus frugilegus*) (41) use the Crossing route. To cross the Sea of Japan in the shortest way, the rooks fly from their wintering sites up to the Hokkaido mainland along the Japanese archipelago, turn west–northwest and cross the sea, and breed on the Eurasian continent. Bird banding surveys by the Yamashina Institute (42) also show that the Daurian redstart (*Phoenicurus auroreus*) and rustic bunting (*Emberiza rustica*) migrate between Primorsky Krai and Japan. In contrast, *I. pavlovskyi-*infested black-faced banting (Table 4) were bred in Japan and migrated to Sakhalin, though *I. pavlovskyi* have not been collected in Sakhalin by flagging vegetation (43).

When the Rishiri and Asahikawa types diverged is unknown as ticks do not have a molecular clock. The evolutionary rate of mitochondrial COI in insects is 2.3%/mya (44). This clock indicates that the Rishiri and Asahikawa types separated approximately 0.45 mya since the nucleotide difference in the complete *I. pavlovskyi* COI gene between Rishiri and Asahikawa types was 1.04% (16/1,359 nt) (Supplement S6). The invertebrate model (1.76 ± 0.66–1.22 ± 0.27/mya) reported by Wilke et al. (45) is another molecular clock. This clock indicates that the haplogroups separated approximately 0.85–0.59 mya. The estimated separation of the haplogroups occurred before Hokkaido, including Rishiri Island, separated from the continent (approximately 12,000 years ago) (46). We assume that the haplogroups did not diverge on the Hokkaido mainland or Rihsiri Island; instead, their ancestral populations separated elsewhere on the continent and then arrived at different times and/or by different routes. This is consistent with the haplotype network (Fig. 4) and the nucleotide difference between the Rishiri and Asahikawa types (16/1,539 nt) being higher than those between the Tomsk and Japanese haplogroups (7/1,539 and 13/1,539 nt for the Rishiri and Asahikawa types, respectively). Further understanding of the trails and population structure of *I. pavlovskyi* in the Far East relies on extensive field surveys of the Eurasian Continent, including that of Primorsky Krai, where a previous study indicated the presence of *I. pavlovskyi* (1, 3).

A survey of wild rodents indicated that *M. rex* and *A. speciosus* (Table 5) were infected with Anaplasmataceae spp. whose 16S rRNA and *groEL* sequences were identical to those of *Ca*. E. khabarensis (Fig. 6). The *Ca*. Ehrlichia khabarensis (taxonomy ID 430555), initially referred to as *Ehrlichia* sp., is a rodent-borne bacterium reported in the Khekhtsir Nature Reserve near Khabarovsk, Russia (27, 28) (Fig. 1). One specific feature of *Ca*. E. khabarensis in rodents is limited to the Khekhtsir Nature Reserve (48°15′N, 135°01′E), and it is absent from other areas in Khabarovsk, as well as various spots including Irkutsk, Novodibirsk, and Sverdlovsk (27). Regardless of extensive field surveys of ticks by flagging vegetation in the Khekhtsir Nature Reserve (28), no tick has been identified as a vector for *Ca.* E. Khabarensis. The survey included predominant *I. persulcatus* adults. The tick species not included in the study, such as less than 3% of the tick population (*I. pavlovskyi*, *I. angustus,* and *Ixodes maslovi*) and/or premature ticks, may participate in the transmission of *Ca.* E. khabarensis. In our study, microorganisms were detected in the host-seeking adult *I. pavlovskyi* (Table 7). Recently, a gene sequence identical to that of *Ca.* E. khabarensis was found in a Great Basin pocket mouse (*Perognathus parvus*）captured in Okanagan, approximately 200 km east of Vancouver, Canada (47). We assume *Ca.* E. khabarensis is possibly transported by migrating birds across not only the Sea of Japan but also the Pacific Ocean. *I. pavlovskyi* has not been reported from the American continent. Thus, *I. pavlovskyi* may only play an important role as a disseminator of tick-borne microorganisms. As emerging infectious diseases caused by *Ehrlichia* spp. have been increasingly reported (48, 49), the transmission dynamics of ticks and birds should be quantified globally.

PCR and sequencing analyses indicated that wild small mammals (9/41) were infected with *Babesia microti* U.S. lineage (Table 5). The lineage is widely distributed in the Northern Hemisphere and genetically further classified into three sub-lineages: North America, Europe–Central Asia, and East Asia, based on marker genes such as *CCT7* and *beta-tubulin* (15, 50). The *CCT7* sequences of the Asian sub-lineage distributed in Korea, Vladivostok, Irkutsk, and eastern Hokkaido (around Nemuro, Fig. 1) varied, whereas *CCT7* from Rishiri Island was identical to those reported in eastern Hokkaido, from *Myodes rutilus, Myodes rufocanus*, and *I. persulcatus* (Fig. 6). In an endemic area of eastern Hokkaido, *I. pavlovskyi* was not collected in our previous studies (15, 51). Examination of molted nymphs and adults (Table 6) showed that *I. pavlovskyi* transstadially transmitted to the US lineage. Previous transmission studies using US lineage and *I. persulcatus* larvae showed that the maximum transmission rate in resulting nymphs was 31.5% (51). Although few ticks were tested in this study, four out of nine ticks were positive (44.4%), indicating that *I. pavlovskyi* has high vectorial capacity.

## Data Availability

Accession numbers of the *I. pavlovskyi* complete mitochondrial sequence are LC578482 (Rishiri-type) and LC633335 (Asahikawa-type). Other accession numbers are listed in Supplements S3 and S4.

## SUPPLEMENTAL MATERIAL

Supplemental material msphere.00213-23-s0001.pdf Supplements S1 to S8.

ASM does not own the copyrights to Supplemental Material that may be linked to, or accessed through, an article. The authors have granted ASM a non-exclusive, world-wide license to publish the Supplemental Material files. Please contact the corresponding author directly for reuse.
