# Supplementary material for: The presence of Ixodes pavlovskyi and I. pavlovskyi–borne microorganisms in Rishiri Island: an ecological survey
Source: mSphere. 2023 Nov 6;8(6):e00213-23. doi: 10.1128/msphere.00213-23 (PMC10871164; doi:10.1128/msphere.00213-23)
Supplement: Supplemental material — Supplements S1 to S8. [file msphere.00213-23-s0001.pdf]

Supplement 1: Phylogenetic tree of *I. pavlovskyi* and *I. persulcatus*

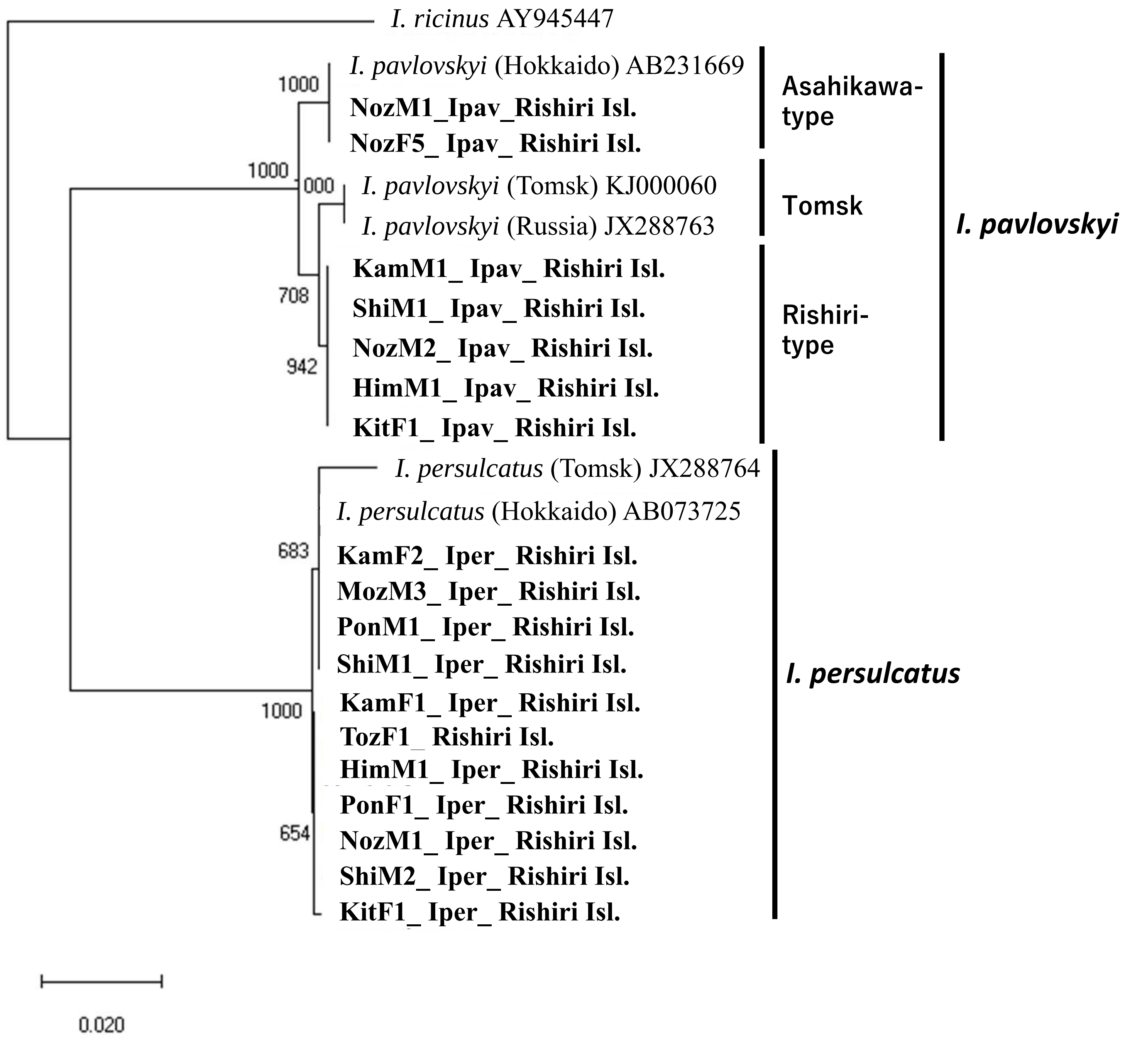

The phylogenetic tree based on *Cytochrome c oxidase subunit I* (*COI*) gene sequences was constructed using the NJ method. Ticks collected on Rishiri Island are shown in bold. Bootstrap values based on 1000 replications are shown at each branch. (Zamoto-Niikura et al. 2020 Rishiri studies 39. 41-46, modified with permission).

S2. Primers used in this study.

Target, reference sequence and primer	Direction	1st/2nd	Sequence 5'-3'	Position	Reference
Mitochondria, *Ixodes* spp. (*Ixodes pavlovskyi* mitochondrion, NC_023831)					
Ixodes COI F1	Forward		GARAGAGGAGCAGGRACAGGATGAAC	1579-1604	in this study
Ixodes COI R1	Reverse		GTGTAATTGATCCTATAATTGCAAATACAGCTCC	2374-2407	in this study
IxoMt_18F	Forward		AAGCTAGTGGGTTCATACCCCAAATATGA	20-50	in this study
IxoMt_8326F	Forward		ACATTTTAAAACGATAAATTCCATAAATTCCTAA	8560-8690	in this study
IxoMt_3458R	Reverse		GGTTTAAGAGACCAGTGCTTAAATATTTCAGCCA	3510-3540	in this study
IxoMt_9356R	Reverse		GTACATAAATTTTGGATATTTAAAGAGATTATC	9640-9670	in this study
Cytochrome b, mammals (*Homo sapiens* Mitochondrion NC_012920)					
L14724	Forward		CGAAGCTTGATATGAAAAACCATCGTTG	14697-14724	Irwin et al. 1991
H15915	Reverse		AACTGCAGTCATCTCCGGTTTACAAGAC	15915-15942	Irwin et al. 1991
Anaplasmataceae 16S rRNA (*Candidatus* Ehrlichia khabarensis strain m3, KR063138)					
E16S-5F	Forward	1st	AGAACGAACGCTRGCGGYAAGC	(-)2-20	Tabara et al. 2007
E16S-3R	Reverse	1st	ACCCYAGTCACYVACCCMACC	1412-1432	Tabara et al. 2007
E16S-200F	Forward	2nd	GATCAGCCACACTGGAACTGAGA	273-295	Tabara et al. 2007
E16S-1162R	Reverse	2nd	CATTGTAGCACGTGTGTAGCCCA	1162-1184	Tabara et al. 2007
Anaplasmataceae groEL (*Can* E. khabarensis strain m3, KR063139)					
gro607F	Forward	1st	GAAGATGCWGTWGGWTGTACKGC	236-258	Tabara et al. 2007
gro1249R	Reverse	1st	AGMGCTTCWCCTTCWACRTCYTC	728-750	Tabara et al. 2007
gro667F	Forward	2nd	ATTACTCAGAGTGCTTCTCARTG	386-408	Tabara et al. 2007
gro1121R	Reverse	2nd	TGCATACCRTCAGTYTTTTCAAC	923-945	Tabara et al. 2007
Bartonella taylorii gltA (strain Far East I, AY584852)					in this study
B.taylorii_gltA-F1	Forward	1st	CATTGATGGTAATAAAGGAATATTGCTTTATCGTG	35-69	in this study
B.taylorii_gltA-F2	Forward	2nd	GCTTTATCGTGGTTATCCTATTGACCAACTGGCTG	59-93	in this study
B.taylorii_gltA-R1	Reverse	1st and 2nd	GGTTGGGAATCCTAAGGCTTTTAATGTAATGCCAG	953-992	in this study
Piroplasminda 18SrRNA (*Bab. microti* strain RI, XR_001160977)					
Piro 0F	Forward	1st	GCCAGTAGTCATATGCTTGTGTTA	15-38	Tsuji et al. 2006
Piro6R	Reverse	1st	CTCCTTCCTYTAAGTGATAAGGTTCAC	1687-1714	Tsuji et al. 2006
Piro 1F	Forward	2nd	CCATGCATGTCTWAGTAYAARCTTTTA	48-74	Tsuji et al. 2006
Piro5.5R	Reverse	2nd	CCTYTAAGTGATAAGGTTCACAAAACTT	1694-1720	Tsuji et al. 2006
Babesia microti US-lineage *CCTeta* (*Bab. microti* strain GI, AB362581)					
UScct-1	Forward	1st	GGTGTGCCAGGCAAATGCCATTTTAAG	1-27	Nakajima et al.
UScct-4	Reverse	1st	GGTGTGAAAATAATAGGACTTTATGTA	2210-2235	Nakajima et al.
UScct-2	Forward	2nd	GCGATAAATCATACAGTTGTATCAC	37-61	Nakajima et al.
UScct-3	Reverse	2nd	ATTAACAAAACATCCTGCGTTAGTCA	2243-2269	Nakajima et al.

S3. *COI* sequence of *I. pavlovskyi* and *I. persulcatus* , determined in this study

Species	Haplogroup	Haplotype	Accession number
Ixodes persulcatus		H2	LC745074
		H5	LC745075
		H6	LC745076
		H7	LC745077
		H8	LC745078
		H9	LC745079
		H10	LC745080
		H11	LC745081
		H12	LC745082
		H13	LC745083
		H14	LC745084
		H15	LC745085
		H16	LC745086
		H17	LC745087
		H18	LC745088
		H19	LC745089
Ixodes pavlovskyi	Rishiri-type	H20	LC745090
		H21	LC745091
		H22	LC745092
		H23	LC745093
	Asahikawa-type	H24	LC745094
		H25	LC745095
		H26	LC745096
		H27	LC745097
		H28	LC745098

S4. Accession numbers

	Wild rodent		*Candidatus* Ehrlichia khabarensis		*Bartnella tayrolii*		*Babesia microti*	
ID	Species	Cyt-b	*16S rRNA*	*GloEL*	*16S rRNA*	*gltA*	*18S rRNA*	*CCT7*
2020 October collected								
RIS01	*A. speciosus*	identical to LC743913						
RIS02	*A. speciosus*	identical to LC743913	identical to LC744904	identical to LC744905				
RIS03	*M. rex*	LC743911	identical to LC744904	identical to LC744905				
RIS04	*M. rex*	LC743912			LC744913	LC744906		
RIS05	*A. speciosus*	LC743913						
RIS06	*M. rex*	identical to LC743914	LC744904	LC744905				
RIS07	*M. rex*	LC743914						
RIS08	*A. speciosus*	identical to LC743913	identical to LC744904	identical to LC744905			LC744901	LC744903
RIS09	*A. speciosus*	LC743920						
RIS10	*A. speciosus*	LC743921						
RIS11	*A. speciosus*	LC743922					identical to LC744901	identical to LC744903
RIS12	*A. speciosus*	identical to LC743913	identical to LC744904	identical to LC744905				
RIS13	*M. rex*	LC743915	identical to LC744904	identical to LC744905				
RIS14	*Sorex unguiculatus*	ND					identical to LC744901	identical to LC744903
RIS15	*A. speciosus*	LC743923						
RIS16	*A. speciosus*	LC743924						
RIS17	*A. speciosus*	LC743925						
RIS18	*A. speciosus*	LC743926						
RIS19	*A. speciosus*	LC743927						
RIS20	*A. speciosus*	identical to LC743913						
RIS21	*M. rex*	LC743916						
RIS22	*A. speciosus*	LC743928						
RIS23	*A. speciosus*	LC743929						
RIS24	*A. speciosus*	LC743930						
RIS25	*M. rufocanus bedfordiae*	LC743902	identical to LC744904	identical to LC744905				
RIS26	*M. rufocanus bedfordiae*	LC743903			identical to LC744913	LC744907		
RIS27	*M. rufocanus bedfordiae*	LC743904			identical to LC744913	LC744908		
RIS28	*M. rufocanus bedfordiae*	LC743899			identical to LC744913	LC744909		
RIS29	*M. rufocanus bedfordiae*	LC743900			identical to LC744913	LC744910		
RIS30	*M. rufocanus bedfordiae*	LC743905	identical to LC744904	identical to LC744905				
RIS31	*M. rufocanus bedfordiae*	LC743901						
RIS32	*A. speciosus*	LC743931						
RIS33	*Sorex unguiculatus*	ND						
2022 May collected								
RIS 22-1	*M. rufocanus bedfordiae*	LC743910						
RIS 22-2	*M. rex*	LC743917			identical to LC744913	LC744912	identical to LC744901	identical to LC744903
RIS 22-3	*M. rufocanus bedfordiae*	LC743909					identical to LC744901	identical to LC744903
RIS 22-4	*M. rufocanus bedfordiae*	LC743907					identical to LC744901	identical to LC744903
RIS 22-5	*M. rex*	LC743918					identical to LC744901	identical to LC744903
RIS 22-6	*M. rufocanus bedfordiae*	LC743906			identical to LC744913	LC744911	identical to LC744901	
RIS 22-7	*M. rufocanus bedfordiae*	LC743908					identical to LC744901	identical to LC744903
RIS 22-8	*M. rex*	LC743919					identical to LC744901	identical to LC744903

Supplement 5A

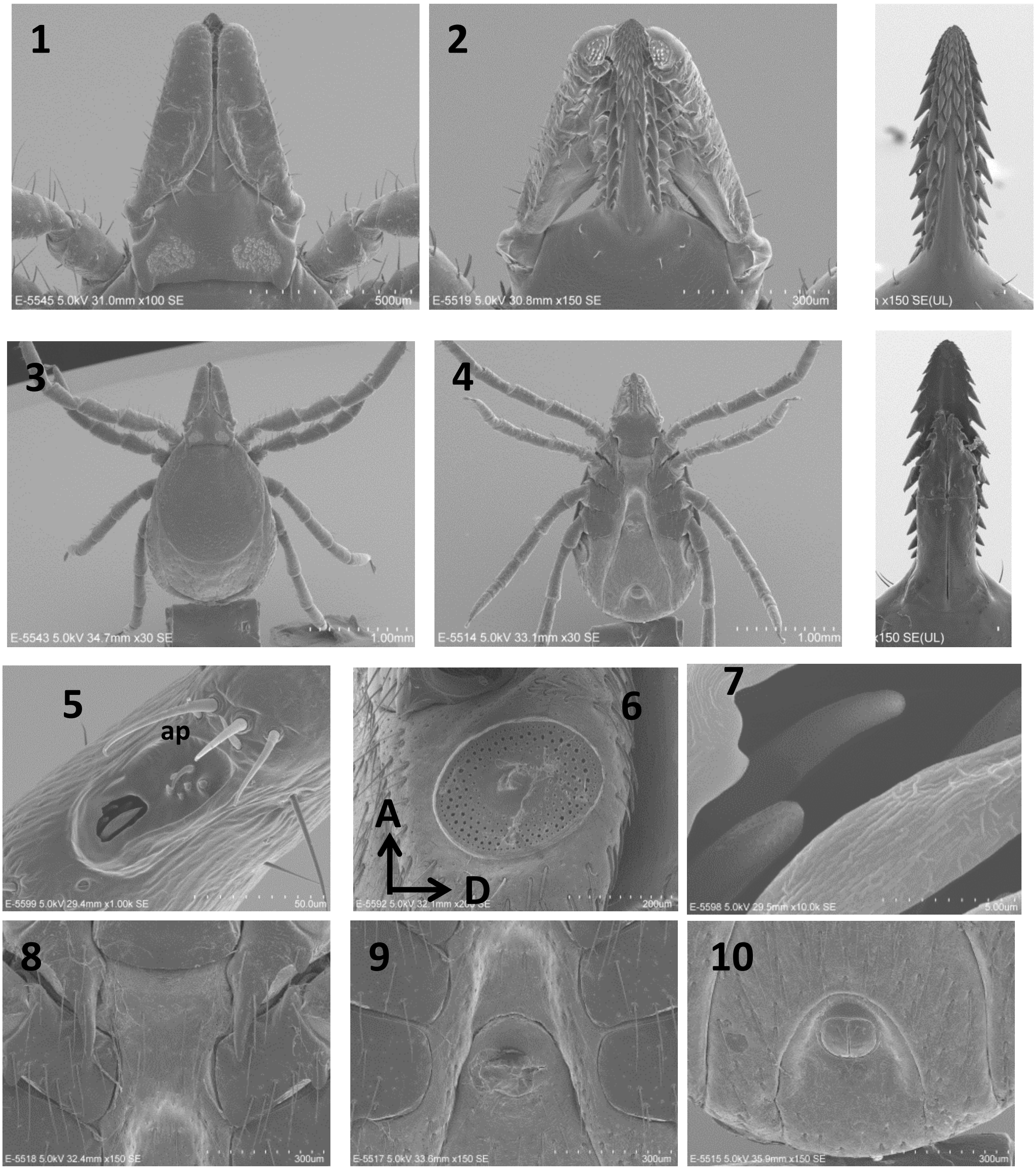

S5A. *Ixodes pavlovskyi* female (Rishiri-type) 1. Capitulum, dorsal. 2. Capitulum, ventral. 3. Idiosoma dorsal. 4. Idiosoma ventral.5. Haller's organ. 6. Spiracular plate. 7. capsule of Haller's organ 8. Coxa I. 9. genital apparature 10. anal groove. Abbreviations: ap, anterior pit (sensillas); A , anterior; D, dorsal

S5

Supplement 5B

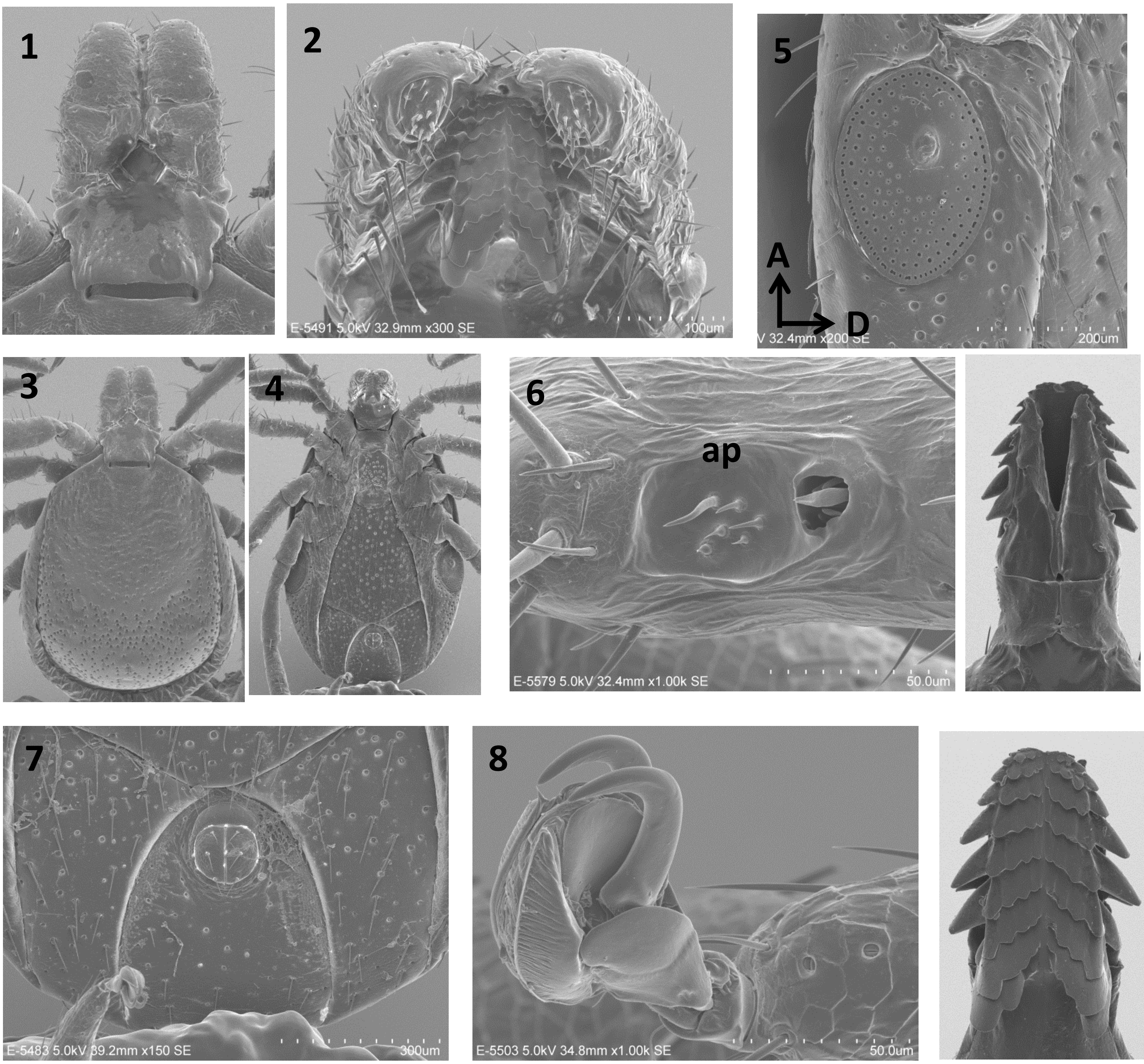

S5B. *Ixodes palrovskyi* male (Rishiri-type) 1. Capitulum, dorsal. 2. Capitulum, ventral. 3. Idiosoma dorsal. 4. Idiosoma ventral.5. Spiracular plate. 6. Haller's organ. 7. anal groove 8. pretarsus. Abbreviations: ap, anterior pit (sensillas); A , anterior; D, dorsal

S6. Sequence identity and difference count of complete mitochondrial genomes (14,575nt)

	Asahikawa-type	Rishiri-type	Tomsk
Asahikawa-type LC633335	ID	142	255
Rishiri-type LC578482	99.00%	ID	189
Tomsk KJ000060	98.20%	98.70%	ID

Sequence identity and difference count of complete mitochondrial *COI* sequences (1,539 nt)

	Asahikawa-type	Rishiri-type	Tomsk
Asahikawa-type LC633335	ID	16	13
Rishiri-type LC578482	98.90%	ID	7
Tomsk KJ000060	99.10%	99.50%	ID

S7

Pictures of *Ixodes pavlovskyi* feeding on wild animals

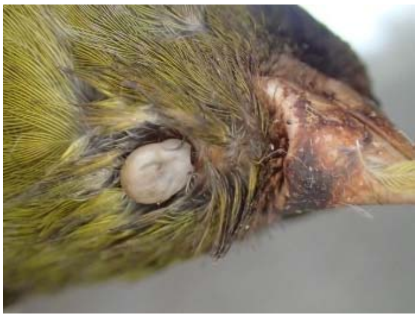

Oriental greenfinch*
(*Chloris sinica*)

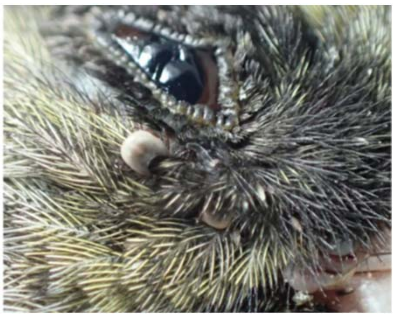

Black-faced bunting
(Emberiza spodocephala)

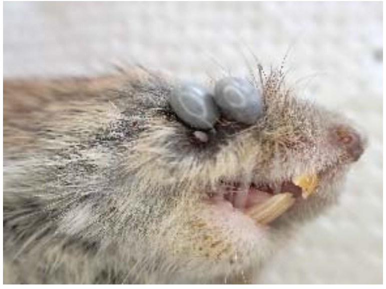

Myodes rex (RIS22-2)

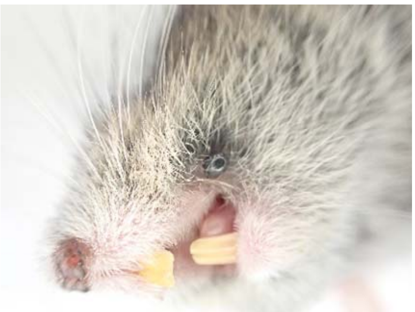

M. rufocanus bedfordiae
(RIS22-3)

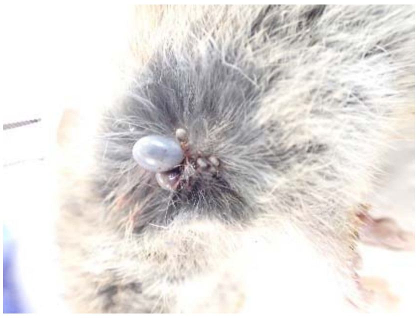

M. rex (RIS22-5)

* Sato et al. Rishiri Studies (40): 25–28, 2021, reproduced with permission.

S8

Accession numbers of *toll* gene

LC777299–LC777319
